# Terminal branching pattern of posterior tibial artery in the tarsal tunnel: a cadaveric study with its clinical importance in medial ankle surgeries

**DOI:** 10.1007/s00276-026-03914-x

**Published:** 2026-06-03

**Authors:** Nashra Khan, Chandni Gupta, Rahul Shekhar, Suriti Pathak, Shivam Thaker, Mayank Jha, Simki Mehta, Souhardya Das, Benzeer Idli

**Affiliations:** 1 Kasturba Medical College, Manipal Academy of Higher Education, Manipal, India; 2 Department of Anatomy, Kasturba Medical College, Manipal Academy of Higher Education, Manipal, 576104 India

**Keywords:** Posterior tibial artery, Tarsal tunnel, Dellon–McKinnon line, Anatomical variation, Medial ankle surgery

## Abstract

**Purpose:**

The posterior tibial artery (PTA) branching within the tarsal tunnel is subject to significant anatomical variation, which can complicate surgical procedures such as tarsal tunnel decompression, flap harvesting, and medial ankle fixation. This study aimed to quantify the morphometry of the PTA bifurcation relative to the Dellon–McKinnon malleolar-calcaneal line (DML) in order to define vascular “safe zones.”

**Methods:**

Twenty-five formalin-fixed adult cadaveric limbs were dissected. The bifurcation of the PTA into the medial and lateral plantar arteries was identified. Measurements were taken in cm with the help of digital vernier calipers and angle with the help of goniometer. The following measurements included the bifurcation level relative to the DML (superior to, at, or inferior to), the distance from the DML, the bifurcation angle, and total foot length. The SSPS software was used for data analysis. Descriptive statistics were conducted for all the parameters. Correlation analysis was done to check for any association between foot length and artery branching length and artery branching angle. Spearman’s Rank Correlation was done to check for the correlation between foot length and the medial and lateral plantar artery lengths. To establish whether there is any difference in foot length, angle of bifurcation, and distance of bifurcation from DML between limbs whose PTA divides superior to and inferior to the DML we have used an independent samples t- test.

**Results:**

The PTA bifurcated superior to the DML in 52%, at the level of the DML in 28%, and inferior to the DML in 20% of specimens. The mean bifurcation angle was 22.60°. A strong positive correlation was found between foot length and angle of division below the DML line, but it was not significant. (r = 0.516, *p* value-0.374). Rare variations, including arterial trifurcation and early anastomosis, were documented.

**Conclusion:**

The majority of PTA bifurcations occur proximal to or at the level of the DML. This high prevalence of proximal branching suggests that surgical incisions for medial ankle exposure are statistically safer when positioned at or distal to the DML to avoid iatrogenic arterial trauma.

## Introduction

Arising as one of the terminal branches of the popliteal artery, the posterior tibial artery (PTA) plays a crucial role in perfusing the plantar aspect of the foot. Within the confines of the tarsal tunnel, the PTA bifurcates into medial and lateral branches accompanied by the posterior tibial nerve and its divisions. Precise comprehension of the ‘ morphometry and branching patterns is indispensable for surgical interventions involving the medial aspect of the foot and ankle, including tarsal tunnel decompression, flap reconstruction, bypass surgery, and various orthopedic or endoscopic procedures [3, 11, 17].

In embryonic life, the PTA arises from the femoral and arterial systems, following the remodeling of the primitive sciatic and popliteal vessels during limb bud morphogenesis [10]. Developmental anomalies, including the persistence or regression of embryonic vascular pathways, influence both the site of bifurcation and the orientation of branching [16]. Such variations can deviate the course and level of branching of the PTA with respect to the DML. The DML is an imaginary anatomical reference line stretching between the center of the medial malleolus to the center of the calcaneal tuberosity. As a consistent anatomical guide, the DML facilitates accurate localization of the PTA bifurcation and delineates ‘safe zones’ for incisions and dissections [6].

According to prior anatomical studies, the bifurcation of the PTA occurs superior to the DML in 50–70% of cases, along the line in 20–30%, and inferior to it in 10–20%. This reflects significant inter-limb and inter-individual variability [7, 8, 12]. This variability underscores the importance of validating morphometric data across distinct populations, as ethnic and anatomical factors can influence vascular branching characteristics. The present study aimed to analyze terminal branching morphology within the tarsal tunnel, evaluate its spatial orientation relative to the DML, and explore morphometric relationships between foot length and the angle of bifurcation. By refining anatomical understanding, the outcomes of the study aim to improve operative precision and lower the potential for iatrogenic vascular and neural trauma during medial ankle and foot surgeries.

## Materials and method

### Study design and study participants

This cross-sectional morphometric investigation involved 25 formalin-preserved adult cadaveric limbs of indeterminate sex, with ages ranging from 30 to 75 years.

The limbs were selected from body donors (Mean age = 52.5 years., N = 25) had been fully embalmed in the formalin solution by the licensed bequest program, Manipal, India. Specimens were examined over a two-week period, following approval from the Institutional Ethics Committee (IEC no. 695–2025), which ensured adherence to ethical standards and the preservation of dignity throughout data collection.

Sample size was calculated using effect size 0.6, power 80%, error 0.05 and 5% significance.

Using the formula

$$ {\text{n }} = {\text{ z2 X }}\sigma {2}/{\mathrm{d2}} $$

where, n = Sample needed, z = Value of normal standard distribution, ϭ = Standard deviation, d = Absolute precision.

### Inclusion and exclusion criteria

Specimens exhibiting fractures, significant deformities, or pre-existing impairments to deep neurovascular structures pertinent to this study (specifically, the PTA) were excluded. Only adult feet were included.

### Dissection method and data collection

The limbs underwent a preliminary inspection for any observable gross defects or fractures. Those limbs that satisfied the inclusion criteria were segregated and properly labeled. Data pertaining to the medial aspect of the foot and the measurement of foot length (from the medial malleolus to the tip of the great toe) were systematically recorded onto the data sheets.

The feet were anatomically positioned, with the ankles dorsiflexed to 90 degrees. Dissection of the feet was done according to the guidelines of Cunningham’s Manual of Practical Anatomy [9]. An oblique incision measuring 45 degrees and 3 cm was executed on the skin and soft tissue over the medial malleolus. Subsequent retraction of the skin and soft tissue was performed, and the oblique incision was extended until the periosteum of the medial malleolus and the attachment site of the flexor retinaculum were identified. Further blunt dissection was carried out to detach the soft tissue from the flexor retinaculum; thereafter, skin incisions were made perpendicular to the initial oblique incision at both ends to establish a viewing window. The flexor retinaculum was then detached from its anchorage on the tibia and reflected to reveal the components of the tarsal tunnel. Dissection hooks were employed to delineate the tibialis posterior, flexor digitorum longus, and flexor hallucis longus tendons from the tarsal tunnel, thereby exposing the posterior tibial nerve and artery. Upon visualization of the neurovascular bundle, dissection proceeded parallel to the trajectories of the nerve and artery, both proximally and distally, to determine the bifurcation point. Following sufficient exposure to the point of bifurcation, morphometric assessments were conducted.

**Fig. 1:**
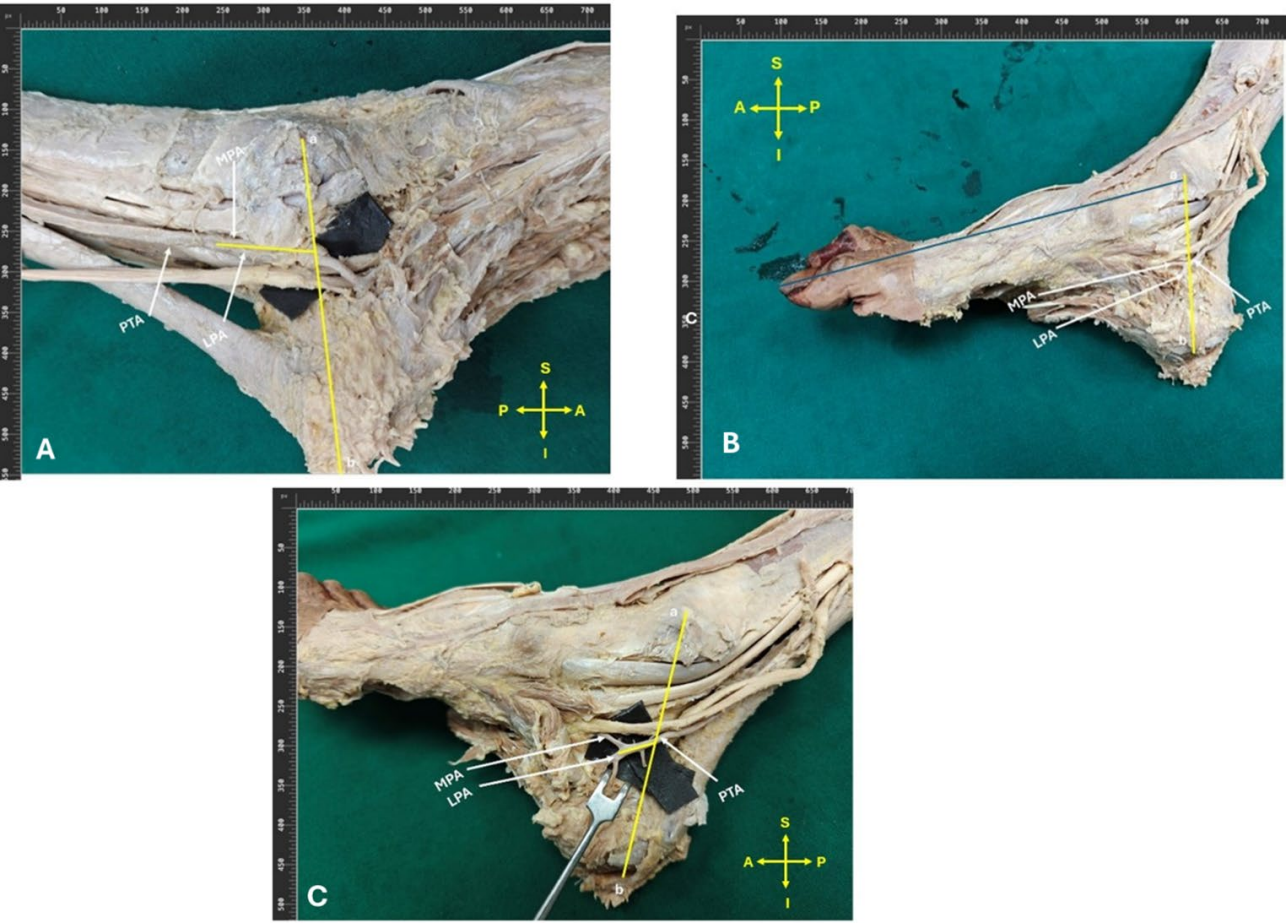
The Dellon–McKinnon malleolar-calcaneal line (DML) was used for measurements and morphometric analysis of the bifurcation point of the Posterior Tibial artery (PTA). The DML is defined as an imaginary line that connects the center of the medial malleolus to the center of the calcaneal tuberosity. Foot length was also measured from medial malleolus to the tip of the first digit (“great toe”). The following parameters were measured: **a** the positioning of the bifurcation in relation to the line (Superior, Inferior, or at the level of the line), **b** if positioned Superior or Inferior to the DML, the specific distance from it, and (**c**) the angle at which the bifurcation occurs. **a**. Medial malleolus, **b**. Calcaneum, **c**. first digit (great toe), PTA, posterior tibial artery; LPA, lateral plantar artery; MPA, Medial plantar artery

The measurements and morphometric analysis of the bifurcation point were evaluated in relation to the Dellon–McKinnon malleolar-calcaneal line (DML). The DML, which involves the deltoid ligament of the ankle, has both clinical and surgical importance. It is defined as an imaginary line that connects the center of the medial malleolus to the center of the calcaneal tuberosity. (Fig. 1) The parameters measured encompassed: (a) the positioning of the bifurcation in relation to the line superior to the line (type 1), at the level of the line (Type 2) and inferior to the line (Type 3), (b) if positioned superior to or inferior to the DML, the specific distance from it, and (c) the angle at which the bifurcation occurs. A measurement was also taken from the medial malleolus to the tip of the first digit (“great toe”), which was considered foot length. The measurements are depicted in (Fig. 1) To avoid inter-observer variation and maintain uniformity, each parameter was measured twice, and the mean value was taken.

### Statistical analysis

The SSPS software was used for data analysis. Descriptive statistics were conducted for all the parameters. Comparison of right and left parameters were done using unpaired T- Test. Comparative Analysis of the morphometric parameters and the division of the Artery with Respect to DML was also done using ANOVA for 3 groups and unpaired T-Test for 2 groups. Correlation analysis was done to check for any association between foot length and artery branching length and artery branching angle. Spearman’s Rank Correlation was done to check for the correlation between foot length and the medial and lateral plantar artery lengths. To establish whether there is any difference in foot length, angle of bifurcation, and distance of bifurcation from DML between limbs whose PTA divides superior to and inferior to the DML we have used an independent samples t- test.

## Results

A total of 25 cadaveric limbs were assessed in the study, of which 17 were left and 8 were right limbs.

### Location of PTA with relation to DML

**Fig. 2:**
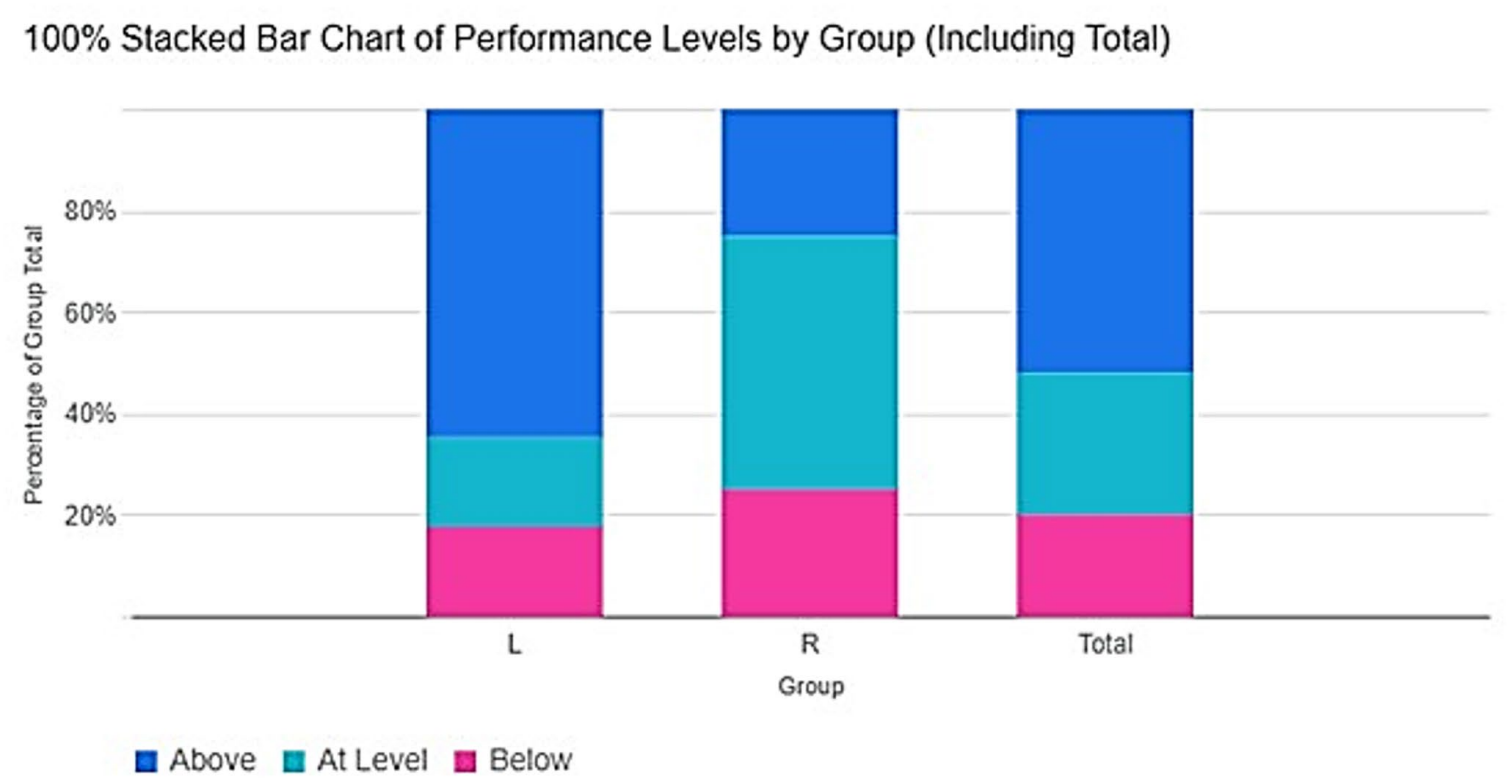
Stacked bar chart showing the distribution of the site of bifurcation based on side of limb and in total. In the left limb, most of the arteries were found to divide superior to the DML line (64.71%). This contrasts with the right limb, which showed a greater division at the level of the DML line (50%) than superior to (25%) or inferior to the line (25%). L, Left; R, Right

From the 25 specimens that were assessed, with regard to the division of the posterior tibial artery into the medial and lateral plantar arteries, 52% (the majority) divided superior to the DML line (type 1), while 28% (type 2) and 20% (type 3) divided at the level and inferior to the DML line, respectively. In the left limb, most of the arteries were found to divide superior to the DML line (64.71%). This contrasts with the right limb, which showed a greater division at the level of the DML line (50%) than superior to (25%) or inferior to the line (25%). (Fig. 2) (Table 1).

**Table 1:** Site of the division of the PTA with respect to the DML. In the left limb, most of the arteries were found to divide superior to the DML line

	Artery Division with respect to DML			Total
	Above (%)	At Level (%)	Below (%)	
L	11 (64.71)	3 (17.65)	3 (17.65)	17
R	2 (25)	4 (50)	2 (25)	8
Total	13 (52)	7 (28)	5 (20)	25

This contrasts with the right limb, which showed a greater division at the level of the DML line than superior or inferior to the line

### Morphometric data related to PTA

Table 2 contains morphometric data regarding limb side. The mean foot length was 19.58 ± 1.48 cm, n = 25. The mean angle at the division of the artery was 22.60° ± 14.45°. Comparison of right and left side measurements are also done which shows no significance foot length (*p* value-0.272), artery length above and below (*p* value-0.088) and artery angle (*p value*-0.305). (Table 2) Morphometric parameters of PTA in relation to DML are shown in Table 3. When artery divides above the DML Foot length was 19.07 ± 1.09 cm, artery length above DML was 3.62 ± 1.55 cm and artery angle was 21.38° ± 15.39°.

**Table 2:** A detailed summary of the morphometric parameters of the Left and the Right Foot with their comparison using unpaired T test

	Side/Measurement	Count	Mean ± SD	Minimum	Maximum	Unpaired T-test (*P* value)
MM to Toe (Foot length) (cm)	L	17	19.72 ± 1.83	17.3	25.2	0.272
	R	8	19.29 ± 1.14	17	20.6	
Artery Length above/below (cm)	L	17	2.56 ± 1.97	0	6.5	0.088
	R	8	1.39 ± 1.92	0	5	
Artery Angle (°)	L	17	23.71° ± 17.7°	7	55	0.305
	R	8	20.25° ± 11.21°	7	40

**Table 3:** A detailed summary of the morphometric parameters of the division of the Posterior tibial artery (PTA) with relation to Dellon–McKinnon malleolar-calcaneal line (DML)

	Side/Measurement	Count	Mean ± SD	Minimum	Maximum
Above	MM to Toe (Foot length) (cm)	13	19.07 ± 1.09	17.3	21.5
	Artery Length above/below (cm)	13	3.62 ± 1.55	1	6.5
	Artery Angle (°)	13	21.38° ± 15.39°	7	51
At level	MM to Toe (Foot length) (cm)	7	19.81 ± 1.39	17	21.3
	Artery Length above/below (cm)	7	0 ± 0	0	0
	Artery Angle (°)	7	17.43° ± 11.31°	10	40
Below	MM to Toe (Foot length) (cm)	5	20.6 ± 2.69	18.5	25.2
	Artery Length above/below (cm)	5	1.49 ± 0.95	0.3	2.7
	Artery Angle (°)	5	33° ± 18.07°	10	55

### Comparison of PTA data in relation to division of the artery with respect to DML and with foot length

While comparing morphometric parameters and the division of the Artery with Respect to DML it was noted that artery length with respect to division of the Artery superior to and inferior to DML was significant (*P* value: 0.0059). It was also noted that artery angle with respect to division of the Artery below and at DML was significant (*P* value: 0.0473). (Table 4) While comparing various artery morphometric features, including angle at division and length superior to or inferior to the DML line, with foot length, at various sites of division, no significant correlation was found. A strong negative correlation was found between foot length and artery division length inferior to the DML line (r = − 0.824) with no statistical significance (*p* value-0.087). A strong positive correlation was found between foot length and angle of division inferior to the DML line (r = 0.516), but it was not significant (*p* value-0.374). The results are depicted in Table 5.

**Table 4:** Comparative Analysis of the Morphometric parameters and the division of the Artery with Respect to Dellon–McKinnon malleolar-calcaneal line (DML). For comparing 3 groups ANOVA is done and for comparing 2 groups unpaired T test is done

	ANOVA (Above/At-Level/Below DML) (*P* value)	Unpaired T-Test (Above vs. Below)(*P* value)	Unpaired T- Test (Above vs. At Level) (*P* value)	Unpaired T-Test (Below vs. At Level) (*P* value)
MM to Toe (Foot length)	0.278	0.138	0.076	0.260
Artery length	–	0.0059*	–	–
Artery angle	0.211	0.279	0.0946	0.0473*

^*^P value < 0.05 is significant

**Table 5:** Correlation between artery division morphometry and foot length at respective levels of division which show no significant difference at any level as all *p* values were > 0.05

	Parameters being correlated	N	Pearsons correlation(r)	*p*-value
Above	Foot Length & Artery Length Above	13	− 0.166	0.588
	Foot Length & Division Angle	13	0.04	0.897
At level	Foot Length & Division Angle	7	0.135	0.772
Below	Foot Length & Artery Length Below	5	− 0.824	0.087
	Foot Length & Division Angle	5	0.516	0.374

### Anatomical variation in relation to PTA and its branches

**Fig. 3:**
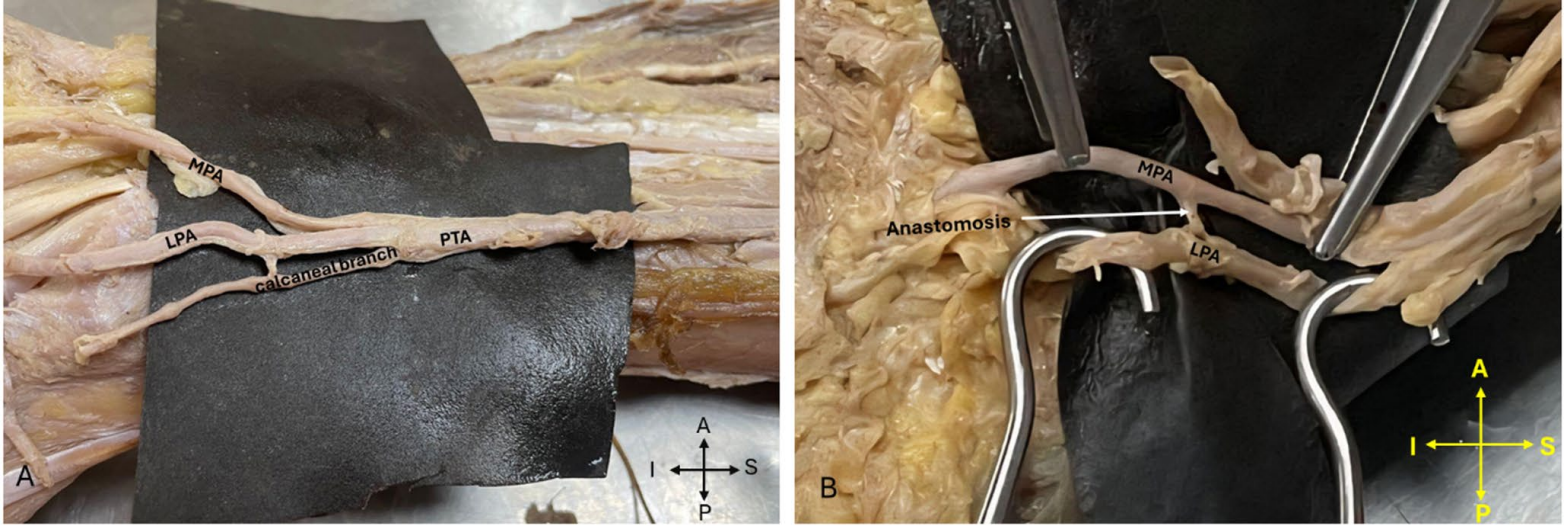
**A**. Showing the foot where there were three terminal branches of PTA: two plantar arteries and one calcaneal branch, **B**. Showing the foot where there was a presence of anastomosis between the medial and lateral plantar arteries. PTA, posterior tibial artery; LPA, lateral plantar artery; MPA, Medial plantar artery

While looking for anatomical variations it was noticed that in one of the cadaveric limbs there were three terminal branches of PTA: two plantar arteries and one calcaneal branch. Additionally, we also noted in two limbs the presence of anastomosis between the medial and lateral plantar arteries within 3 cm of the site of terminal branching (Fig. 3).

## Discussion

A detailed understanding of the terminal branching of the PTA is vital for tarsal tunnel surgery to prevent inadvertent arterial injury and its associated ischemic complications. The current study establishes that a predominant proximal bifurcation pattern relative to DML makes surgical incisions positioned at or below the line safer for operative exposure of the medial ankle. This observation aligns with established surgical guidelines, which recommend that entry points be located 1–1.5 cm below DML to avoid damage to the PTA bifurcation zone [6, 11, 17].

### Location of PTA with relation to DML

In this study classification of PTA was done in relation to DML. DML also forms the superior boundary of Kagers triangle. Kagers triangle is bound by three points (1) tip of the medial malleolus, (2) center of the calcaneus, and (3): tuberosity of the navicular bone. It helps define the anatomical region where neurovascular structures (posterior tibial nerve, posterior tibial artery) travel to the foot [15]. 3 types were classified- 1) superior to the DML (Type 1) in 52% of cases, 2) At the level of the line (Type 2) in 28% and 3) inferior to it (Type 3) in 20%. The distribution of bifurcation levels in this study parallels that reported by Dalmia et al. and Hussain et al. who noted bifurcation of the artery superior to the DML in 54–60% of cases and level bifurcation in 25% of cases [7, 12]. The dominance of bifurcation of the artery superior to the DML corresponds to adaptive morphogenic variations along the tibial-fibular axis, influenced by embryologic remodeling dynamics and functional mechanical stresses of the adult foot [10, 16].

### Morphometric data related to PTA

The mean bifurcation angle was 23.7°, aligning with previously reported ranges of 20–30° documented in cadaveric studies [7, 13]. Statistical analysis demonstrated the absence of a significant relationship between foot length and bifurcation level (*p* value-0.588 for superior to the DML; *p* value-0.087 for inferior to the DML) or the bifurcation angle (*p* value-0.897 superior to; *p* value-0.374 inferior to). These results suggest that the branching level is determined by developmental and vascular remodeling rather than limb proportions.

### Anatomical variation in relation to PTA and its branches

Anatomical variations in bifurcation pattern and site may alter the orientation and length of the vascular pedicle, thereby affecting flap viability. Radiological and Doppler investigations further from the previous study verify that anatomical variability of the PTA may alter the feasibility and results of endovascular access and tibial bypass surgeries [4].

During embryogenesis, the regression of the sciatic arterial system coincides with the formation of the femoral-popliteal axis, with the PTA emerging as a distal offshoot of the tibial-peroneal trunk, its development modulated by local hemodynamic forces. Aberrant persistence or involution of embryonic vascular channels can lead to atypical proximal or distal bifurcation patterns of the PTA [16, 18]. Comparative anatomical studies have revealed comparable variations in bifurcation levels, suggesting a universal morphological variation spectrum [8, 14].

Vascular territories may be jeopardized after trauma or resections, and a clear understanding of the anatomical variations and anastomotic connections in the foot is mandatory for successful reconstruction [5]. In this study we also noted in two limbs the presence of anastomosis between the medial and lateral plantar arteries within 3 cm of the site of terminal branching.

Medial plantar artery (MPA) fasciocutaneous flap has proven to have long-term acceptable results, particularly for small- to medium-sized wounds of the hindfoot, as it preserves sensation to the plantar skin [1]. It has also been reported to be the good choice for the ankle reconstruction, because of the concealed donor site, durable skin, flexible pedicle and reliable blood supply [2]. But the dissection for the fasciocutaneous flap can be tough and needs careful surgical technique to avoid damaging the MPA and the likelihood of necrotic sequelae [1]. So, the origin of MPA from PTA and its distance from medial malleolus will help the surgeons in planning surgeries.

The results of this study will provide essential, precise anatomical measurements that enable surgeons to navigate safely, avoiding critical neurovascular structures during reconstructive surgery, including medial plantar and calcaneal flap construction, vascular graft harvesting, and PTA-based perforator flap procedures [4, 13].

The strength of this study include that this is the first study to quantify the Dellon–McKinnon line variability relative to foot length. This study will provide essential, precise anatomical measurements that enable surgeons to navigate safely, avoiding critical neurovascular structures during percutaneous techniques. So, this study has translational focus on ligamentous reconstruction and minimally invasive surgery (MIS).

The limitation of the present study includes that as a cadaveric study; the morphometry provides a static anatomical map that may not fully reflect the dynamic neurovascular changes and pressure gradients observed in live patients with clinical tarsal tunnel syndrome. This study utilized formalin-fixed cadavers, which may induce tissue shrinkage, potentially underestimating distances. This study did not correlate the bifurcation angle with foot arch height, which may influence artery tension.

## Conclusion

This cadaveric study identifies that the PTA most commonly bifurcates superior to the Dellon–McKinnon line. The results emphasize that the PTA maintains a consistent anatomical course, while its terminal branching shows morphometric diversity. This study might provide a platform for future studies where these Merging cadaveric data with advanced imaging techniques could enhance the accuracy of surgical safe-zone mapping.

## Data Availability

All data supporting the findings of this study are available within the paper and its Supplementary Information.
